# Early acute kidney injury and transition to renal replacement therapy in critically ill patients with SARS-CoV-2 requiring veno-venous extracorporeal membrane oxygenation

**DOI:** 10.1186/s13613-023-01205-x

**Published:** 2023-11-24

**Authors:** Kevin Roedl, Silvia De Rosa, Marlene Fischer, Josephine Braunsteiner, Christian Schmidt-Lauber, Dominik Jarczak, Tobias B. Huber, Stefan Kluge, Dominic Wichmann

**Affiliations:** 1 Department of Intensive Care Medicine, University Medical Centre Hamburg-Eppendorf, Martinistraße 52, 20246 Hamburg, Germany; 2 Centre for Medical Sciences, CISMed, University of Trento, Via S. Maria Maddalena 1, 38122 Trento, Italy; 3 Anesthesia and Intensive Care, Santa Chiara Regional Hospital, APSS, Trento, Italy; 4 III. Department of Medicine, University Medical Centre Hamburg-Eppendorf, Hamburg, Germany; 5 Research Center On Rare Kidney Diseases (RECORD), University Hospital Erlangen, Erlangen, Germany

**Keywords:** Acute kidney injury, ECMO, Fluid overload, Renal replacement therapy, SARS-COV-2

## Abstract

**Background:**

Critically ill patients with severe acute respiratory syndrome coronavirus 2 (SARS-CoV-2) requiring veno-venous extracorporeal membrane oxygenation (vv-ECMO) are at risk for acute kidney injury (AKI). Currently, the incidence of AKI and progression to kidney replacement therapy (RRT) in critically ill patients with vv-ECMO for severe COVID-19 and implications on outcome are still unclear.

**Methods:**

Retrospective analysis at the University Medical Center Hamburg-Eppendorf (Germany) between March 1st, 2020 and July 31st, 2021. Demographics, clinical parameters, AKI, type of organ support, length of ICU stay, mortality and severity scores were assessed.

**Results:**

Ninety-one critically ill patients with SARS-CoV-2 requiring ECMO were included. The median age of the study population was 57 (IQR 49–64) years and 67% (*n* = 61) were male. The median SAPS II and SOFA Score on admission were 40 (34–46) and 12 (10–14) points, respectively. We observed that 45% (*n* = 41) developed early-AKI, 38% (n = 35) late-AKI and 16% (*n* = 15) no AKI during the ICU stay. Overall, 70% (*n* = 64) of patients required RRT during the ICU stay, 93% with early-AKI and 74% with late-AKI. Risk factors for early-AKI were younger age (OR 0.94, 95% CI 0.90–0.99, *p* = 0.02) and SAPS II (OR 1.12, 95% CI 1.06–1.19, *p* < 0.001). Patients with and without RRT were comparable regarding baseline characteristics. SAPS II (41 vs. 37 points, *p* < 0.05) and SOFA score (13 vs. 12 points, *p* < 0.05) on admission were significantly higher in patients receiving RRT. The median duration of ICU (36 vs. 28 days, *p* = 0.27) stay was longer in patients with RRT. An ICU mortality rate in patients with RRT in 69% (*n* = 44) and in patients without RRT of 56% (*n* = 27) was observed (*p* = 0.23).

**Conclusion:**

Critically ill patients with severe SARS-CoV-2 related ARDS requiring vv-ECMO are at high risk of early acute kidney injury. Early-AKI is associated with age and severity of illness, and presents with high need for RRT. Mortality in patients with RRT was comparable to patients without RRT.

**Supplementary Information:**

The online version contains supplementary material available at 10.1186/s13613-023-01205-x.

## Background

In 2019, the emergence of severe acute respiratory syndrome coronavirus 2 (SARS-CoV-2) has spread all over the world [1]. While clinical presentation ranges from mild respiratory symptoms to severe acute respiratory failure in about 40% of patients with COVID-19 admitted to the intensive care unit (ICU) [2–4], SARS-CoV-2 may be accompanied by multiorgan failure and subsequently death in severe cases [5–7]. In selected patients who develop progressive acute respiratory failure refractory to optimal support with conventional mechanical ventilation, the use of veno-venous extracorporeal membrane oxygenation (vv-ECMO) may be considered as therapy option [8, 9].

Early referral to ECMO centres as well as early initiation of vv-ECMO has been proven to be beneficial in these patients [10–12]. Thus, the use of vv-ECMO has increased substantially in critical care units during the last decade [13]. Even though SARS-CoV-2 primarily targets the respiratory system, other organs such as the kidneys may be affected [14, 15]. Although acute kidney injury (AKI) in patients with ECMO is reported in 26–85% of patients [16], the pooled incidence rate of severe AKI in patients with ECMO is 45% [17]. The variation in reported incidences might be attributable to different clinical settings and patient characteristics, but also different definitions for AKI were used [16].

SARS-CoV-2 AKI is associated with disease severity and might be an indicator of poor prognosis [18, 19]. Multiple pathogenic mechanisms of SARS-CoV-2 AKI have been proposed including direct tubular damage, lung-kidney crosstalk, cytokine storm, hypercoagulability, rhabdomyolysis, hypoperfusion and impact of mechanical ventilation as well as inhaled nitric oxide on renal function [20–25]. Despite just one study reported the incidence of severe AKI stages (II/III) in 38% of SARS-CoV-2 [26], 58–60% required the initiation of renal replacement therapy (RRT) and higher rates of RRT in non-survivors were observed [27, 28]. To date, detailed characteristics on the development of AKI and requirement of RRT in patients receiving vv-ECMO due to SARS-CoV-2 has not been reported.

The present study aims to investigate the incidence of early AKI and progression to renal replacement therapy (RRT) in SARS-CoV-2 patients receiving vv-ECMO.

## Methods

### Study population, design and ethics

We retrospectively analyzed consecutive SARS-CoV-2 patients admitted to ICU of the Department of Intensive Care Medicine at the University Medical Center Hamburg Eppendorf (Germany) between March 1st, 2020 and July 31st, 2021. The study was approved by the Ethics Committee of the Hamburg Chamber of Physicians (No.: 2021-300112-WF). Owing to the retrospective character of the study and anonymized data collection, the need for informed consent was waived.

### Inclusion and exclusion criteria

We included all consecutive adult patients (≥ 18 years) with confirmed and symptomatic COVID-19 requiring vv-ECMO support admitted to our department during the study period. Confirmed SARS-CoV-2 was defined as at least one positive result on reverse transcriptase polymerase chain reaction (PCR) obtained from nasopharyngeal swabs and/or bronchial secretions and typical symptoms including dyspnea, fever or cough. Patients without confirmed COVID-19, ongoing ICU stay at the time of data censoring and patients aged < 18 years were excluded.

### Data collection

Patient data were collected from the department’s electronical patient data management system (PDMS, Integrated Care Manager® (ICM), Version 9.1–Draeger Medical, Luebeck, Germany). The data included age, gender, body mass index, comorbidities, admission diagnosis, length of ICU stay, organ support (mechanical ventilation, ECMO, vasopressor support, RRT), medication and laboratory test results.

### Clinical definitions and patient management

Severity of illness was evaluated with the sequential organ failure assessment (SOFA) [29] and the simplified acute physiology II (SAPS II) [30] scores. The Charlson Comorbidity Index (CCI) [31] was calculated for all patients. Clinical management was performed according to national and international guidelines, including prone positioning in moderate to severe ARDS and, restrictive fluid management following the initial resuscitation period [32]. ARDS was defined according to the Berlin definition, using the PaO_2_/FiO_2_ ratio (Horowitz index) as marker for severity [33]. Vasopressor support was initiated to maintain a mean arterial pressure (MAP) of 65 mmHg or higher using norepinephrine [9, 32]. Patients with severe hypoxemic and/or hypercapnic respiratory failure in combination with severe respiratory acidosis refractory to adjunctive therapies received vv-ECMO. Criteria for the initiation of vv-ECMO support were based on the guidelines of the American Thoracic Society (ATS), national recommendations and the EOLIA trial [10, 32, 34]. Prone positioning during vv-ECMO therapy was initiated in patients with persistent severe hypoxemia. The anticoagulation on vv-ECMO was performed using continuously applicated unfractionated heparin. The effect of heparin was monitored using the activated clotting time during the cannulation till start with heparin. The targeted activated thromboplastin time was 40 to 50 s in all patients. AKI and AKI Stage was diagnosed using urine output and/or serum creatinine, based on Kidney Disease: Improving Global Outcomes (KDIGO) criteria [35, 36].

Early-AKI was defined as development of any KDIGO-AKI Stage within the first 72 h after ICU admission, Late-AKI was defined as development of any AKI stage after 72 h in the ICU and with no AKI within the first 72 h. Baseline serum creatinine was defined as the first measured creatinine in the ICU. Indication to start RRT, based on the most recent Austrian/German recommendations [37, 38], was performed by the attending intensivist in accordance with local standardized protocols in patients with severe metabolic acidosis (pH < 7.2), anuria unresponsive to fluids resuscitation measures, hyperkalemia (serum potassium concentration exceeding 6.5 mmol per liter), serum creatinine concentration above 3.4 mg per deciliter, presence of clinically significant organ edema (e.g., pulmonary edema) or uremic complications [37, 38]. RRT in patients with vv-ECMO was performed via a separate central venous access. In general, RRT was performed continuous. Intermittent RRT was performed when patients were stabilized according to local practice procedures. Percentage of fluid overload was calculated via the following: [(cumulative fluid balance–day 3 (liters)—cumulative urinary output–day 3 (liters))/ICU admission weight (kg)] × 100] [39, 40]. Patient survival was assessed at ICU discharge, after 28 and after 90 days. Last day of follow-up was October 1st, 2021.

### Statistical analysis

Data are presented as absolute numbers and relative frequency or median with interquartile range (IQR). Categorical variables were compared via Chi-Square test or Fisher’s exact test, as appropriate. Continuous variables were compared via Mann–Whitney *U*-test. Survival function estimates were calculated using Kaplan–Meier method and were compared by log rank test. To assess factors associated with early-AKI we performed a logistic regression analysis. Clinically relevant variables (Age, BMI, Gender, SAPS II, CCI) were included in the initial model and were eliminated stepwise backwards. We performed an exploratory analysis. Statistical analysis was conducted using IBM SPSS Statistics Version 24.0 (IBM Corp., Armonk, NY). The study protocol was prepared in accordance with the Strengthening the Reporting of Observational studies in Epidemiology (STROBE) recommendations [41].

## Results

**Fig. 1:**
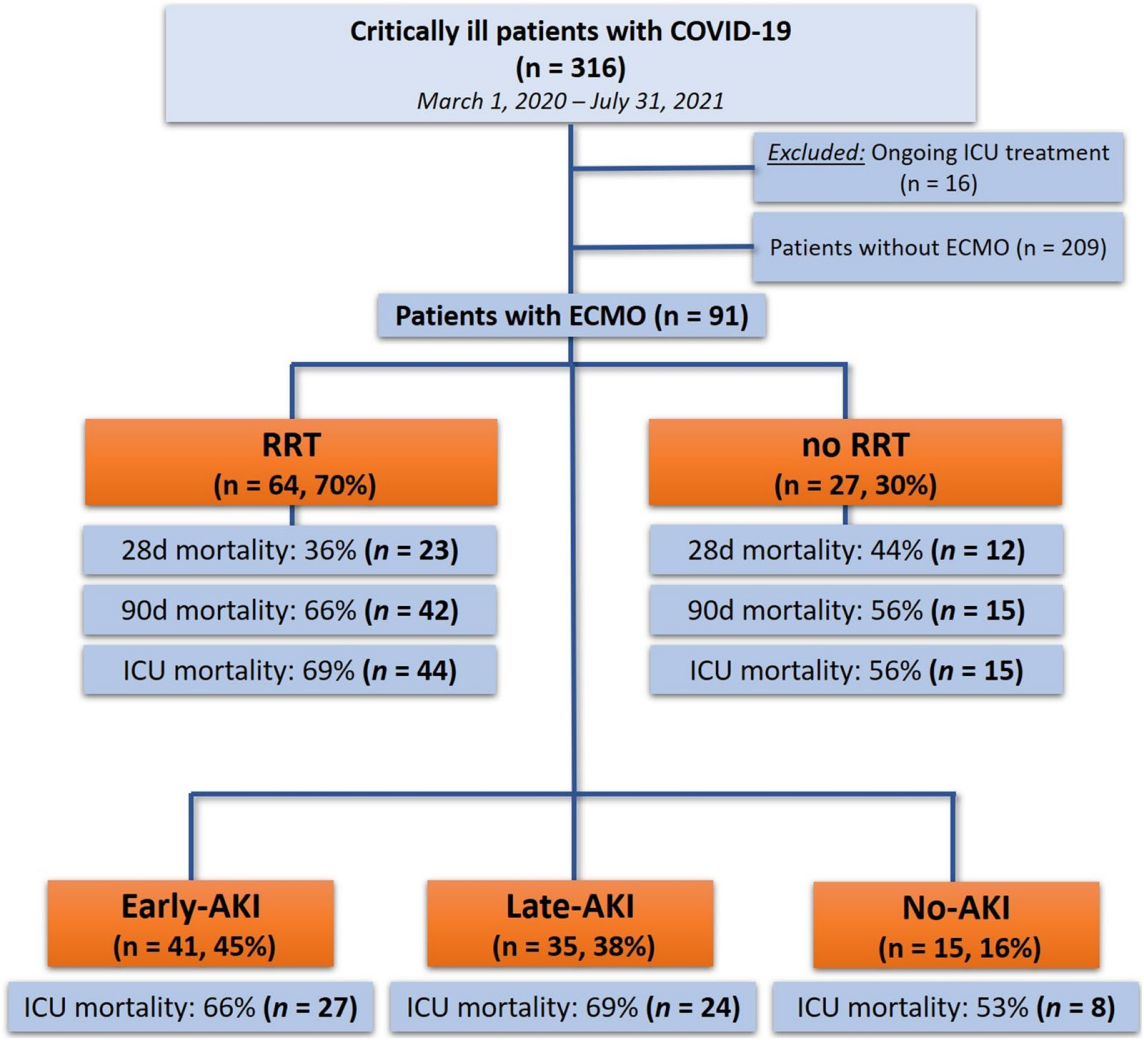
Study flowchart

Overall, 316 critically ill patients with confirmed SARS-CoV-2 infection were admitted to our department during the period from March 1st, 2020 until July 31st, 2021. Sixteen patients with ongoing treatment at the end of the study period were excluded. Of the remaining 300 patients, 91 (30%) received vv-ECMO treatment and were included in the final analysis (see Flowchart Fig. 1).

### Baseline demographic characteristics

Baseline demographic characteristics are reported in Table 1. The median age of patients was 57 (IQR: 49–64) years, 67% (n = 61) were male and the median BMI was 31.7 (27.3–36.2) kg/m^2^. Severity of illness represented by SAPS II was 40 (34–46) and SOFA 12 (10–14) points on admission. The Charlson comorbidity index (CCI) was 1 (0–2) points. The most common comorbidities were arterial hypertension (45%, *n* = 41), diabetes mellitus type II (30%, *n* = 27) and chronic lung disease (18%, *n* = 16). Patients were transferred from other hospitals (88%, n = 80), the peripheral ward (9%, n = 8) or the emergency department (3%, *n* = 3). The median duration of hospital and ICU stay was 37 (19–63) and 33 (16–57) days, respectively.

**Table 1:** Baseline demographic characteristics

Variables	All (*n* = 91)
Age (years)	57 (49–64)
Males	61 (67)
BMI (kg/m^2^)	31.7 (27.3–36.2)
Disease severity (admission)	
SAPS II (pts.)	40 (34–46)
SOFA (pts.)	12 (10–14)
Comorbidities	
Charlson comorb. index, pts	1 (0–2)
Arterial hypertension (*n*, %)	41 (45)
Chronic kidney disease (*n*, %)	2 (2)
Coronary heart disease (*n*, %)	7 (8)
Congestive heart failure (*n*, %)	4 (4)
Diabetes mellitus (*n*, %)	27 (30)
Chronic lung disease (*n*, %)	16 (18)
Smoking (*n*, %)	11 (12)
Admission from	
Transfer from peripheral ward	8 (9)
Transfer from emergency dep	3 (3)
Transfer from other hospital	80 (88)
Outcome	
Duration ICU stay (days)	33 (16–57)
Duration hospital stay (days)	37 (19–63)
ICU mortality	59 (65)
28-day mortality	35 (38)
90-day mortality	57 (63)

Data are expressed as *n* (%) or median (interquartile range)

*BMI* body mass index, *pts* points, *SAPS* simplified acute physiology score, *SOFA* sequential organ failure assessment, *ICU* intensive care unit

### Occurrence of early-, late- and no-AKI

Within the first 72h after ICU admission, 45% (*n* = 41) of the patients developed early-AKI. Patients with early-AKI had AKI stage I (4%, *n* = 4), stage II (4%, *n* = 4) and stage III (36%, *n* = 33). Thirty-five (38%) patients developed late-AKI and fifteen (16%) no-AKI.

Patients with AKI had similar age, male gender and BMI compared to patients with late-AKI and no AKI, as shown in Table 2. However, the severity of illness assessed by SAPS II and SOFA score on admission was significantly higher in patients with early-AKI (Table 2). All patients received vasopressor treatment and were mechanically ventilated. Complications during the ICU stay were frequent, we observed the occurrence of pulmonary embolism only in patients with early (5%) and late-AKI 20% (*p* = 0.03), cardiac arrest occurred in 29% with early-AKI, in 31% with late-AKI and 27% without AKI (*p* = 0.94). Blood gas analysis, urine output as well as fluid balance differed significantly between all groups. Detailed clinical characteristics are shown in Table 2.

**Table 2:** Clinical characteristics of patients with early, late and no acute kidney injury

Variables	Early AKI (*n* = 41)	Late AKI (*n* = 35)	No AKI (*n* = 15)	*p*-value
Age (years)	57 (49–61)	56 (50–65)	60 (47–67)	0.55
Males	31 (76)	22 (63)	8 (53)	0.23
BMI (kg/m^2^)	33.2 (28.7–38.1)	30.8 (26.1–34.9)	29.3 (26.8–33.3)	0.15
Charlson comorb. index, pts	1 (0–2)	1 (0–1)	0 (0–1.5)	0.23
Disease severity				
SAPS II (pts.)	45 (36–55)	36 (31–41)	38 (34–42)	< 0.001
SOFA—admission (pts.)	14 (12–16)	10 (7–12)	12 (11–12)	< 0.001
SOFA—24h (pts.)	15 (13–16)	11 (8–12)	11 (10–12)	< 0.001
ICU procedures				
Vasopressors	41 (100)	35 (100)	15 (100)	1
High Flow-Nasal-Cannula	13 (32)	16 (46)	6 (40)	0.45
Non-Invasive Ventilation	17 (41)	17 (49)	8 (53)	0.69
Mechanical ventilation	41 (100)	35 (100)	15 (100)	1
Renal Replacement Therapy	38 (93)	26 (74)	0 (0)	< 0.001
COVID-19 Therapy				
Remdesivir	5 (12)	5 (14)	3 (20)	0.76
Dexamethasone	28 (68)	30 (86)	13 (87)	0.13
Plasma-Exchange	0 (0)	1 (3)	1 (7)	0.30
Tocilizumab	1 (2)	1 (3)	1 (7)	0.72
Other Antibody-Therapy	0 (0)	0 (0)	0 (0)	–
ARDS—Management				
Prone positioning	29 (71)	28 (80)	9 (60)	0.33
Neuromuscular blockade	28 (68)	20 (57)	9 (60)	0.59
Inhaled nitric oxide	24 (59)	19 (54)	7 (47)	0.73
Glucocorticoid therapy	38 (93)	32 (91)	12 (80)	0.35
Complications—ICU stay				
Pulmonary embolism	2 (5)	7 (20)	0 (0)	0.03
Deep vein thrombosis	4 (10)	4 (11)	0 (0)	0.41
Cardiac arrest	12 (29)	11 (31)	4 (27)	0.94
Neurologic	21 (51)	11 (31)	7 (47)	0.21
Urine output, fluid balance and blood gas				
Lactate, mmol/l—admission	1.8 (1.2–2.8)	1.4 (0.8–1.7)	1.7 (1.2–2.3)	< 0.01
pH, level—admission	7.30 (7.25–7.36)	7.39 (7.29–7.46)	7.39 (7.25–7.49)	< 0.01
Base excess—admission	− 0.7 (− 4.9–3.9)	4.4 (0.8–8.8)	5.3 (3.0–8.1)	< 0.01
Bicarbonate—admission	23 (20–27)	27 (24–31)	28 (26–30)	< 0.01
Creatinine, mg/dl—admission	1.79 (1.13–2.78)	0.8 (0.6–1.1)	0.83 (0.66–1.25)	< 0.001
Urine output, ml—day 1	455 (50–830)	1250 (690–1970)	1220 (535–1840)	< 0.01
Fluid balance, ml—day 1	1817 (360–3644)	300 (-242–648)	560 (98–2011)	< 0.001
Urine output, ml—day 2	600 (140–1510)	2230 (1523–3143)	2910 (1733–3223)	< 0.001
Fluid balance, ml—day 2	2245 (1141–4273)	722 (216–1743)	977 (183–1667)	< 0.001
Urine output, ml—day 3	183 (63–1042)	2390 (1975–3585)	2790 (2340–3070)	< 0.001
Fluid balance, ml—day 3	1978 (246–3459)	74 (− 391–1212)	540 (-432–1480)	< 0.001
Percentage of Fluid Overload	5.4 (3.0–8.2)	− 0.9 (− 3.3–0.6)	− 0.5 (− 2.3–1.1)	< 0.001
Outcome				
Length of stay—ICU (days)	26 (15–57)	46 (34– 63)	21 (14–48)	0.05
Length of stay—hospital (days)	30 (17–63)	43 (28–59)	26 (15–57)	0.07
28-day mortality	18 (44)	9 (25)	8 (53)	0.12
90-day mortality	27 (66)	22 (63)	8 (53)	0.69

Data are expressed as *n* (%) or median (interquartile range)

*ARDS* acute respiratory distress syndrome, *SOFA* sequential organ failure assessment, *SAPS II* simplified acute physiology score II, *pts.* Points, *ICU* intensive care unit

### Final AKI stage and transition to RRT

Of the patients included, 84% (*n* = 76) developed AKI and 16% (*n* = 15) did not develop AKI. Patients with AKI had AKI-KDIGO stage I (13%, *n* = 10), KDIGO stage II (1%, *n* = 1) and KDIGO stage III (86%, *n* = 65). Overall, 70% (*n* = 64) received RRT during the ICU stay. In patients with early-AKI RRT had to be started in 93% (*n* = 38), in patients with late-AKI 74% (*n* = 26) received RRT during the course of ICU stay. 3% (*n* = 2) had chronic kidney disease prior the ICU stay. In 9% (*n* = 6) RRT was started in the referring center. The median time from ICU admission to start of RRT was 3 (1–9) days. In 34% (*n* = 22) RRT was started within 24h of ICU admission. The median duration of RRT was 21 (7–45) days. In patients discharged alive from the ICU, 50% (*n* = 10) were dialysis dependent at time of ICU discharge.

In all patients RRT was primarily used continuously. RRT was performed as Continous Veno-Venous Hemodialysis (CVVHD) in 95% (*n* = 61) and Hemofiltration (CVVHF) in 36% (*n* = 23) both with high flux hemodiafilter. In 13% (*n* = 8) IRRT was used during the ICU stay. RRT was initiated due to one absolute indication in 78% (*n* = 50) and in 61% (*n* = 39) more than one absolute indication for initiation of RRT was found. Particularly, RRT was started based on fluid overload in 70% (*n* = 45), anuria in 44% (*n* = 28), hyperkalaemia in 44% (*n* = 28) and severe metabolic acidosis in 42% (*n* = 27). For detailed characteristics of RRT modality and indication see Additional file 1: Table S1.

### Clinical differences of patients with and without RRT

Patients with and without RRT were comparable regarding baseline characteristics including age (57 vs. 59 years, *p* = 0.26), male gender (70 vs. 59%, *p* = 0.31) and BMI (32.4 vs. 30.9 kg/m^2^, *p* = 0.09). The SAPS II (41 vs. 37 points, *p* < 0.05) and SOFA score (13 vs. 12 points, *p* < 0.05) on admission and after 24h (14 vs. 11 points, *p* < 0.001) were significantly higher in patients receiving RRT than without RRT. All patients received vasopressor therapy. The use of high flow nasal cannula and non-invasive ventilation was similar in both groups. The adjunctive treatment regarding prone positioning (73 vs. 70%, *p* = 0.77), neuromuscular blockade (64 vs. 59%, *p* = 0.67), inhaled nitric oxide (56 vs. 44%, p = 0.70), glucocorticoid therapy (92 vs. 85%, *p* = 0.31) was not different in the groups. The median duration of MV was 32 (16–56) days in RRT and 21 (12–44) days in patients without RRT (*p* = 0.08). The rate of tracheostomy was higher in patients with RRT (66 vs. 52%, *p* = 0.22). The urine output during the first 3 days was significantly lower and the fluid balance higher in patients receiving RRT. Detailed clinical characteristics are shown in Table 3 and see Additional file 1: Table 2.

**Table 3:** Clinical characteristics of patients with and without kidney replacement therapy

Variables	RRT (*n* = 64)	No RRT (*n* = 27)	p-value
Age (years)	57 (49–62)	59 (51–67)	0.26
Males	45 (70)	16 (59)	0.31
BMI (kg/m^2^)	32.4 (27.6–39.2)	30.9 (26.4–33.9)	0.09
Charlson comorb. index, pts	1 (0–2)	1 (0–2)	0.71
Disease severity			
SAPS II (pts.)	41 (35–51)	37 (34–42)	< 0.05
SOFA—admission (pts.)	13 (10–15)	12 (11–12)	< 0.05
SOFA—24h (pts.)	14 (11–16)	11 (10–12)	< 0.001
ICU procedures			
Vasopressors	64 (100)	27 (100)	1
High Flow-Nasal-Cannula	25 (39)	10 (37)	0.86
Non-Invasive Ventilation	29 (45)	13 (48)	0.80
Mechanical ventilation	64 (100)	27 (100)	1
Respiratory—Management			
Prone positioning	47 (73)	19 (70)	0.77
Neuromuscular blockade	41 (64)	16 (59)	0.67
Inhaled nitric oxide	36 (56)	12 (44)	0.70
Glucocorticoid therapy	59 (92)	23 (85)	0.31
Duration of mechanical ventilation	31 (16–56)	21 (12–44)	0.08
Tracheostomy	42 (66)	14 (52)	0.22
Complications—ICU stay			
Pulmonary embolism	8 (13)	1 (4)	0.20
Deep vein thrombosis	8 (13)	0 (0)	0.05
Cardiac arrest	20 (31)	7 (26)	0.61
Neurologic	23 (36)	16 (59)	< 0.05
Urine output, fluid balance and blood gas			
Lactate, mmol/l—admission	1.5 (0.9–2)	1.7 (1.1–2.2)	0.69
pH, level—admission	7.33 (7.25–7.39)	7.39 (7.29–7.47)	0.06
Base excess—admission	1.8 (− 3.2–7.1)	5.3 (0.9–8.3)	0.08
Bicarbonate—admission	25 (22–29)	28 (24–31)	0.05
Creatinine, mg/dl—admission	1.26 (0.81–2.62)	0.80 (0.62–1.08)	< 0.01
Urine output, ml—day 1	650 (183–1363)	1350 (740–1995)	< 0.01
Fluid balance, ml—day 1	781 (140–2897)	456 (-162–1387)	0.15
Urine output, ml—day 2	1365 (300–2245)	2710 (1733–3130)	< 0.001
Fluid balance, ml—day 2	1606 (533–3372)	1039 (299–1743)	0.05
Urine output, ml—day 3	1040 (93–2768)	2375 (2060–2970)	< 0.01
Fluid balance, ml—day 3	1069 (− 340–2581)	544 (50–1502)	0.56
Outcome			
Length of stay—ICU (days)	36 (17–63)	28 (16–50)	0.27
Length of stay—hospital (days)	38 (21–65)	30 (17–56)	0.23
28-day mortality	23 (36)	12 (44)	0.45
90-day mortality	42 (66)	15 (56)	0.36
ICU mortality	44 (69)	15 (56)	0.23

Data are expressed as *n* (%) or median (interquartile range)

*ARDS* acute respiratory distress syndrome, *SOFA* sequential organ failure assessment, *SAPS II* simplified acute physiology score II, *pts.* Points, *ICU* intensive care unit

### Risk factors for early-AKI

Multivariable regression analysis identified age (OR 0.94, 95% CI 0.90–0.99, *p* = 0.02) and SAPS II (OR 1.12, 95% CI 1.06–1.19, *p* < 0.001) as factors significantly and independently associated with occurrence of early-AKI (Additional file 1: Table 3).

### Outcome of patients with AKI and RRT

In patients with early-AKI, late-AKI and no-AKI we observed a median duration of ICU (26 vs. 46 vs. 21 days, *p* = 0.05) and hospital (30 vs. 43 vs. 26 days, *p* = 0.07) stay. The ICU mortality in patients with early-AKI, late-AKI and no-AKI was 66% (*n* = 27), 69% (*n* = 24) and 53% (*n* = 8), respectively (*p* = 0.58). See Kaplan–Meier Analysis Additional file 2: Fig. S1 and Additional file 3: Fig. S2.

**Fig. 2:**
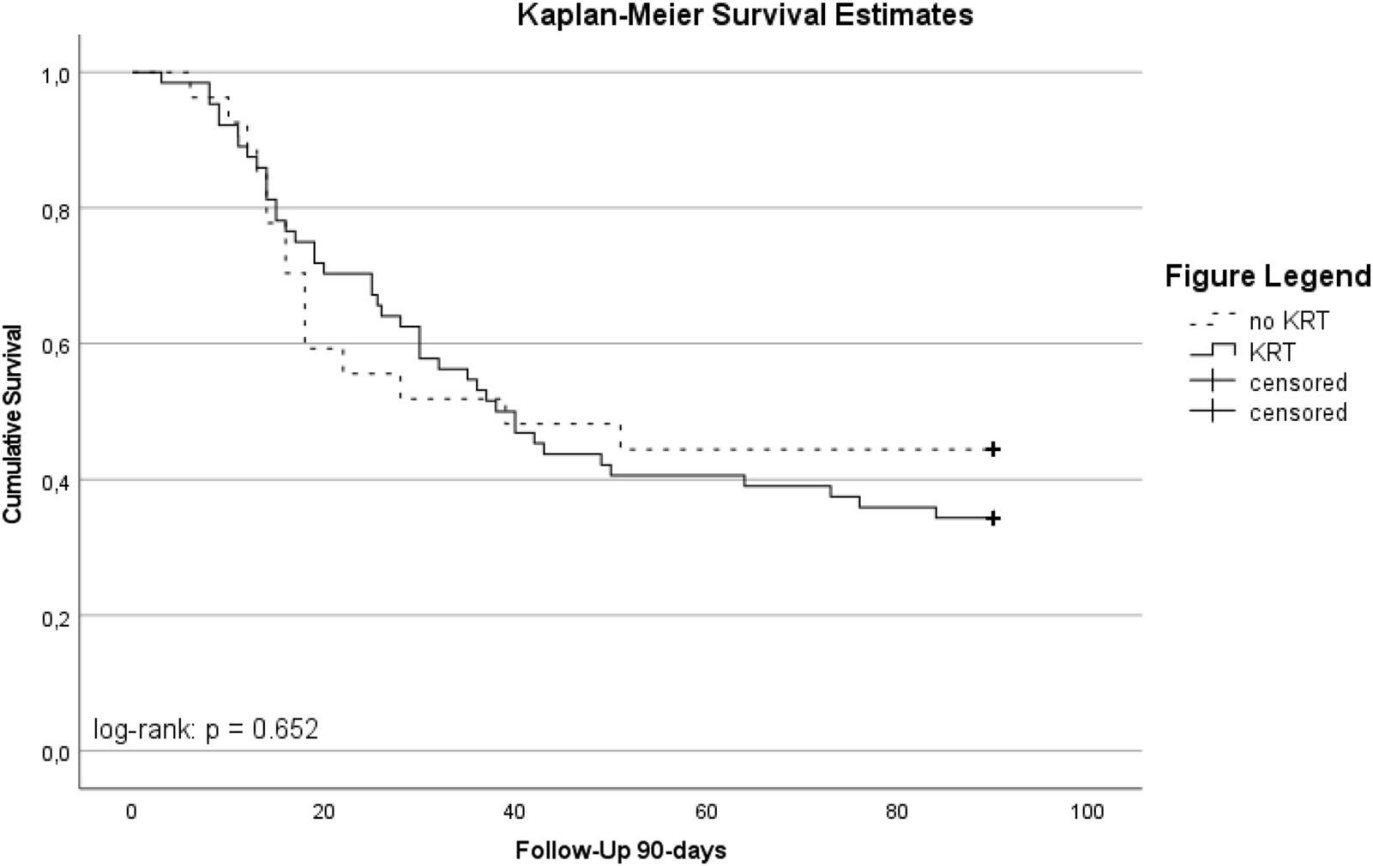
Kaplan–Meier 90-day survival estimates in patients receiving vv-ECMO stratified by the use of kidney replacement therapy

The median duration of ICU (36 vs. 28 days, *p* = 0.27) and hospital (38 vs. 30 days, *p* = 0.23) stay was longer in patients with RRT. A 28-day mortality and 90-day mortality was observed in patients with RRT in 36% (*n* = 23) and 66% (*n* = 44) and in patients without RRT in 44% (*n* = 12) and 56% (*n* = 15), respectively. The Kaplan–Meier survival estimates for 90-day mortality are displayed in Fig. 2.

## Discussion

In the present study, we found that almost half of the patients with SARS-CoV-2 requiring vv-ECMO therapy developed AKI within the first 72h of ICU admission. Further, 70% of patients require RRT during the ICU stay, 50% of patients requiring RRT were dialysis dependent on ICU discharge. Of interest, patients with and without RRT had similar short- and long-term outcomes in spite of different initial severity of illness and complications. To our knowledge, this is the first study focusing exclusively on clinical characteristics and outcomes of patients with early AKI and RRT in critically ill patients with SARS-CoV-2 and vv-ECMO.

The incidence of early AKI in this study of critically ill patients with ARDS was found to be 45%. In critically ill patients, although AKI is a common complication and can be observed in 57% of critically ill patients [42, 43], the occurrence of AKI is associated with high mortality [44, 45]. When focusing on patients with ARDS an incidence of 24 to 44% was reported [46–48]. Although our findings are comparable with that from previous studies, we observed that 84% had AKI during the ICU course when looking at the incidence of AKI during the entire ICU stay. There can be different explanations for this finding. First, all patients had severe ARDS accompanied by severe hypoxemia and requirement of vv-ECMO. Previous studies reported from all stages of ARDS also including less severe form and, therefore, represent a lower severity of illness reasonably accompanied by lower incidence of AKI [47, 49]. Furthermore, all patients in the current cohort were severely ill and had higher SOFA and SAPS II scores on admission compared to other studies [47]. Third, complications like septic shock and cardiac arrest were frequently observed in our cohort. All complications were previously shown to be associated with a high incidence of AKI related to ischemia–reperfusion injury [50, 51]. Fourth, the overall incidence of AKI in patients with ECMO ranges from 26 to 85% [16]. This large difference among studies is mainly attributable to following differences in patient characteristics, the clinical setting and the definition used for detection of AKI. Furthermore, also a changing incidence of AKI during the COVID-19 pandemic, as recently shown in a large critically ill cohort of patients with COVID-19, could be a reason for the heterogeneity of findings across studies [52].

According to recent publications, AKI is more common in patients treated with veno-arterial ECMO than vv-ECMO [17, 53]. The pooled incidence of AKI and the use of RRT in patients with VA-ECMO reaches up to 61% [16, 17]. However, the incidence of the use of RRT in patients with VA-ECMO varies largely in the literature (27–87%) what makes comparison between studies difficult [17]. Compared to the current study in VV-ECMO patients we observed a higher rate of AKI. This may be attributable to different pathophysiological factors regarding VV- and VA-ECMO. Patients with respiratory failure often present with prolonged hypercapnia. Although the respiratory function is supported via VV-ECMO prolonged hypercapnia can induce altered haemodynamics and renal blood flow potentially explaining differences in AKI incidence between patients with VV- and VA-ECMO [54]. Furthermore, a hypercoagulable state due to the non-endothelialised ECMO interface and the destruction of the glycocalyx can cause microemboli and microthrombi [55, 56]. These microemboli and microthrombi in the renal vasculature are particularly found in the patients on VA-ECMO which also explains differences between VV- and VA-ECMO support. Future research should focus on causes of AKI and the pathophysiology regarding different forms of ECMO support.

In our population, we observed a high ICU mortality in patients with early- and late-AKI. Particularly, mortality was delayed in patients with late-AKI, most likely complicating the ICU course via a second hit event. The high mortality in our study population might be attributable to the severity of illness as well as to the high prevalence of sepsis related to superimposed infection during ECMO. The prevalence of hospital-acquired infections during ECMO is 10–12% [57]. Generally, in critically ill patients with SARS-CoV-2 secondary infection rates were reported in 16–45% [58, 59]. Furthermore, patients with SARS-CoV-2 and need for ECMO showed also elevated infection rates up to 58–86%, which was significantly associated with risk for death [60, 61]. Furthermore, there might also be differences in patients selected for ECMO which impact outcome [62].

In general, mortality rates in this cohort are comparable to other studies in a similar cohort [27, 63]. Of interest, we observed that early AKI was significantly associated with need of RRT in the further ICU course. In detail 93% of patients with early AKI required RRT, whereas 74% with late-AKI required RRT in the further ICU course. This highlights the early visibility of kidney alterations and predictive ability of RRT within the first 72h in this cohort.

In general, it has been suggested that AKI is associated with SARS-CoV-2 severity and might be an indicator of poor prognosis. This study exclusively included patients with SARS-CoV-2. It is known that multiple pathogenic mechanisms of SARS-CoV-2 AKI have been proposed including inflammation, cytokine release, possible viral invasion as well as hemodynamic instability, low cardiac output and impact of mechanical ventilation on renal function [20–22]. The high rate of AKI and RRT maybe also be an expression of direct kidney involvement, as previously proposed, or complications during the ICU stay like pulmonary embolism or cardiac arrest [15]. However, it was previously suggested that severe SARS-CoV-2 AKI is tightly intertwined with critical illness and systemic inflammation [14]. Of interest, incidence of SARS-CoV-2 AKI might be higher compared with other types of severe respiratory failure [64]. However, detailed characteristics on development of RRT in patients receiving vv-ECMO due to COVID-19 associated ARDS has not been reported to date.

VV-ECMO has been used as life-saving therapy option for patients with severe respiratory failure. Use of vv-ECMO in patients with ARDS related to viral infections was previously reported during the influenza A (H1N1) pandemic as well as the Middle East respiratory syndrome coronavirus (MERS-CoV) outbreaks [65, 66]. Its use has increased substantially during the past years [13]. The pooled incidence of RRT in patients with ECMO therapy is 45% [17]. In this study, we observed that 70% required RRT throughout the ICU stay. Generally, risk factors for AKI in patients with ECMO are widespread and include older age and pre-existing comorbidities [16]. In patients with ECMO, RRT is mainly initiated to manage or prevent fluid overload, followed by AKI and electrolyte disturbances [16, 67]. Traditional complications of AKI, such as electrolyte derangements, uraemia, and fluid overload are considered to contribute to the poor pulmonary outcomes associated with AKI [48, 63, 68]. In the present cohort, we observed that 78% had an absolute indication for start of RRT and 61% presented more than one indication. The main cause for start of RRT was fluid overload observed in 70% of cases. Our results are in line with evidence present in literature in the paediatric population [69–71]. Further, recent results of a survey regarding the practice of RRT initiation in patients on ECMO showed that fluid overload and anuria were the most prevalent indications [72]. 62% of patients had a positive fluid balance, suggesting that fluid overload and the ability to achieve a negative fluid balance are potentially important therapeutic targets associated with improved survival. One reason for the high percentage of positive fluid balance in the present cohort could be the high incidence of sepsis, which requires large volume resuscitation and is associated with fluid overload. In fact, CRRT provides flexibility and control in fluid management, and has been shown to enhance the ability to achieve negative fluid balance during ECMO.

Of interest, we found that patients with RRT had a higher mortality without reaching statistical significance. Mortality rates of patients with RRT while on ECMO are high and the likelihood of dying for patients receiving RRT was reported to be three times higher than that of those without RRT [17]. It generally remains unclear whether RRT itself directly increases mortality risk or it represents an epiphenomenon of disease severity [16, 73]. The reason why patients with and without need for RRT had the same mortality rate, remains unknown. It could be that there was a change in clinical practice due to evolving therapy options during the pandemic that affected kidney function and requirement of RRT. Further, also initiation strategies of RRT could have had an effect.

We acknowledge the following limitations in our study. First, this was a retrospective study and multiple unmeasured variables may have affected the outcomes. Our conclusions need to be validated by larger, prospective studies in the future. Second, we present the results of a single centre with a high expertise in the management of critically ill patients with ARDS. Our results may not be generalizable to other cohorts. Third, due to the retrospective design, pre-admission kidney function could not be well estimated. Forth, changes in clinical practice over time may have influenced outcomes of critically ill patients with COVID-19. Fifth, a recent consensus report of the “Acute Disease Quality Initiative” workgroup proposed that early AKI is defined as AKI that occurs within 48h as opposed to the used definition of 72h in the current manuscript which could lead to different outcomes in comparison to other scientific articles. Sixth, due to local standards RRT was performed via a separate venous access coming along with inherited side effects with this approach. Seventh, residual confounding is a matter of concern and cannot be entirely excluded.

## Conclusion:

In our study, the incidence of SARS-CoV-2 AKI, based on KDIGO criteria, was 45% within the first 72h of ICU admission with 70% of RRT requirement. Early-AKI is associated with older age and severity of illness, and presents with high need for RRT. Mortality in patients with RRT was comparable to patients without RRT. The fluid overload estimation and monitoring during the 72 h of ICU admission might be helpful in identifying critically ill patients with vv-ECMO support at risk for developing AKI. This warrants further investigation in future larger trials.

### Supplementary Information

**Additional file 1: Table S1.** Indications for initiation of RRT and RRT modalities used. **Table S2.** Pre-existing comorbidities. **Table S3.** Logistic regression model for factors associated with early AKI; Hierarchical stepwise backwards elimination of insignificant variables, change of parameter estimate > 10% = confounding variable. **Table S4.** Initial ECMO parameters in patients with early, late and no acute kidney injury

**Additional file 2: Figure S1.** Kaplan–Meier 28-day survival estimates in patients receiving vv-ECMO stratified by early AKI, late AKI and no AKI.

**Additional file 3: Figure S2.** Kaplan–Meier 90-day survival estimates in patients receiving vv-ECMO stratified by early AKI, late AKI and no AKI.

## Data Availability

The datasets supporting the conclusions of this article are included within the article.
