# Supplementary material for: Early acute kidney injury and transition to renal replacement therapy in critically ill patients with SARS-CoV-2 requiring veno-venous extracorporeal membrane oxygenation
Source: Ann Intensive Care. 2023 Nov 24;13:115. doi: 10.1186/s13613-023-01205-x (PMC10673790; doi:10.1186/s13613-023-01205-x)
Supplement: Supplementary file 1 — Additional file 1: Table S1. Indications for initiation of RRT and RRT modalities used. Table S2. Pre-existing comorbidities. Table S3. Logistic regression model for factors associated with early AKI; Hierarchical stepwise backwards elimination of insignificant variables, change of parameter estimate > 10% = confounding variable. Table S4. Initial ECMO parameters in patients with early, late and no acute kidney injury [file 13613_2023_1205_MOESM1_ESM.docx]

Supplementary Table 1 – Indications for initiation of RRT and RRT modalities used

| *Variables* | *RRT*  *(n = 64)* |
| --- | --- |
| Initiation of RRT Absolute Indication Relative Indication > 1 Criteria for Initiation | 50 (78)  14 (22)  39 (61) |
| Cause for Initiation RRT* Lactate Fluid Overload - Fluid Overload – Present - Fluid Overload – Prevention  Anuria Hyperkaliemia Severe Metabolic Acidosis Uraemia | 29 (45)  45 (70)  36 (56)  9 (14)  28 (44)  28 (44)  27 (42)  0 (0) |
| RRT Modality *Primary RRT Modality* CRRT IRRT  *Overall overview of RRT Modality* Continuous Veno-Venous Hemodialysis Continuous Veno-Venous Hemofiltration IRRT | 64 (100) 0 (0)   61 (95) 23 (36) 8 (13) |

***Data are expressed as n (%) or median (interquartile range),*** **more than one indication per patient possible*
*Abbreviations:* RRT, kidney replacement therapy; CRRT, continuous kidney replacement therapy; IRRT, intermittent kidney replacement therapy;

Supplementary Table 2 – Pre-existing comorbidities

| *Parameters* | *RRT*  *(n = 64)* | *No RRT*  *(n = 27)* | *p*-value |
| --- | --- | --- | --- |
| AIDS *n (%)* | 0 (0) | 0 (0) | 1 |
| Cerebral arterial disease *n (%)* | 4 (6) | 3 (11) | 0.43 |
| Chronic lung disease *n (%)* | 10 (16) | 6 (22) | 0.45 |
| Chronic kidney disease *n (%)* | 2 (3) | 0 (0) | 0.35 |
| Congestive heart disease *n (%)* | 3 (5) | 1 (4) | 0.83 |
| Connective tissue disease *n (%)* | 6 (9) | 2 (7) | 0.76 |
| Coronary heart disease *n (%)* | 6 (9) | 1 (4) | 0.35 |
| Dementia *n (%)* | 0 (0) | 0 (0) | 1 |
| Diabetes Mellitus *n (%)* | 18 (28) | 9 (33) | 0.34 |
| Liver cirrhosis *n (%)* | 0 (0) | 0 (0) | 1 |
| Peripheral arterial disease *n (%)* | 2 (3) | 0 (0) | 0.35 |
| Malignancy *n (%)*  Solid tumor  Leukemia  Lymphoma  Solid tumor with metastases | 2 (3)  2 (3)  0 (0)  0 (0) | 1 (4)  1 (4)  1 (4)  0 (0) | 0.89  0.89  0.12  1 |

*Abbreviations:* AIDS, acquired immune deficiency syndrome; n, number;

Supplementary Table 3 – Logistic regression model for factors associated with early AKI; Hierarchical stepwise backwards elimination of insignificant variables, change of parameter estimate >10% = confounding variable

| *Logistic regression* | Covariables | OR (95% CI) | *p* value |
| --- | --- | --- | --- |
| *Final model* | **Age** (years)  **Gender** (male vs. female)  **SAPS II** (points) | 0.94 (0.90 – 0.99)  0.35 (0.12 – 1.05)  1.12 (1.06 – 1.19) | 0.02  0.06  < 0.001 |

Abbreviations: OR, odds ratio; CI, confidence interval; SAPS II, simplified acute physiology score II; SAPS II – transformation via natural logarithm before inclusion into logistic regression analysis; The initial model included: Age, BMI, Gender, SAPS II, CCI.

Supplementary Table 4 – Initial ECMO parameters in patients with early, late and no acute kidney injury

| *Variables* | *Early AKI*  *(n = 41)* | *Late AKI*  *(n = 35)* | *No AKI*  *(n = 15)* | *p*-value |
| --- | --- | --- | --- | --- |
| ECMO – Management initial  ECMO – flow (l/min)  ECMO – speed (RPM)  Sweep gas flow (l/min)  ECMO – Oxygenation setting (FiO_2_) | 4.0 (3.1 – 4.6)  3345 (3055 – 3978)  3 (2.7 – 3.8)  1.0 (1.0 – 1.0) | 4.5 (4.3 – 5.0)  3648 (3077 – 4188)  3 (2.0 – 3.6)  1.0 (1.0 – 1.0) | 4.0 (3.7 – 4.5)  3700 (3493 – 3808)  3 (2.5 – 4)  1.0 (1.0 – 1.0) | 0.21  0.42  0.44  0.99 |

***Data are expressed as n (%) or median (interquartile range)****Abbreviations:* ECMO, extracorporeal membrane oxygenation
