# Supplementary figures and images for: Early acute kidney injury and transition to renal replacement therapy in critically ill patients with SARS-CoV-2 requiring veno-venous extracorporeal membrane oxygenation
Source: Ann Intensive Care. 2023 Nov 24;13:115. doi: 10.1186/s13613-023-01205-x (PMC10673790; doi:10.1186/s13613-023-01205-x)

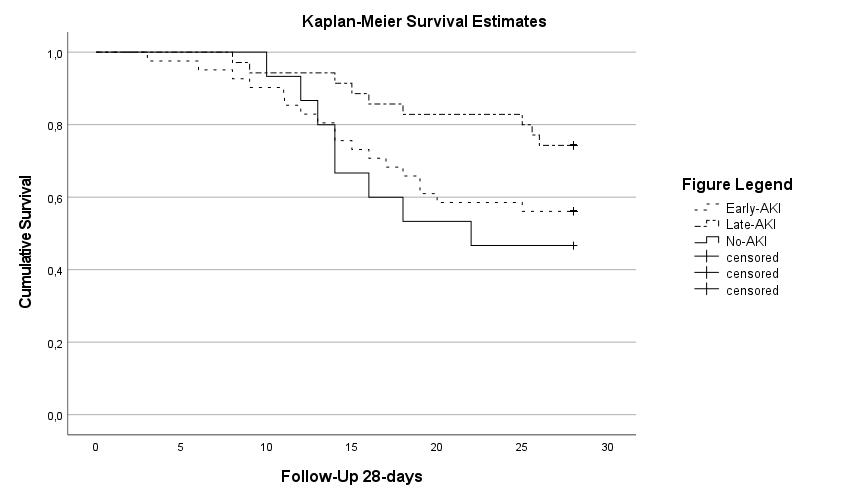

Supplement: Supplementary file 2 — Additional file 2: Figure S1. Kaplan–Meier 28-day survival estimates in patients receiving vv-ECMO stratified by early AKI, late AKI and no AKI. [file 13613_2023_1205_MOESM2_ESM.jpg]

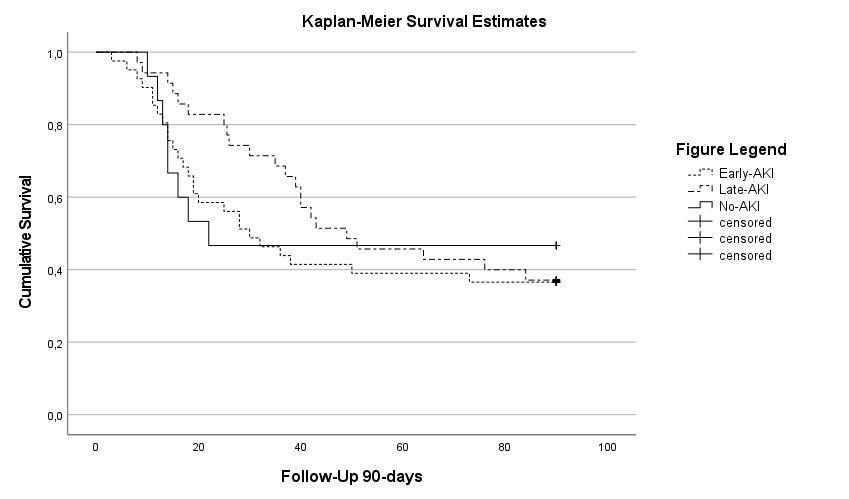

Supplement: Supplementary file 3 — Additional file 3: Figure S2. Kaplan–Meier 90-day survival estimates in patients receiving vv-ECMO stratified by early AKI, late AKI and no AKI. [file 13613_2023_1205_MOESM3_ESM.jpg]
